# Leveraging artificial intelligence to identify high-risk patients for postoperative sore throat: An observational study

**DOI:** 10.17305/bb.2023.9519

**Published:** 2024-06-01

**Authors:** Qiangqiang Zhou, Xiaoya Liu, Huifang Yun, Yahong Zhao, Kun Shu, Yong Chen, Song Chen

**Affiliations:** 1 Department of Anesthesiology, The Affiliated Changzhou Second People’s Hospital of Nanjing Medical University, Changzhou, Jiangsu, China; 2 The Third Affiliated Hospital of Soochow University, Changzhou, Jiangsu, China; 3 Department of Orthopedics, The Quzhou Affiliated Hospital of Wenzhou Medical University, Quzhou People’s Hospital, Quzhou, Zhejiang Province, China

**Keywords:** Random forest (RF), neural network (NN), extreme gradient boosting (XGBoost), postoperative sore throat (POST)

## Abstract

Postoperative sore throat (POST) is a prevalent complication after general anesthesia and targeting high-risk patients helps in its prevention. This study developed and validated a machine learning model to predict POST. A total number of 834 patients who underwent general anesthesia with endotracheal intubation were included in this study. Data from a cohort of 685 patients was used for model development and validation, while a cohort of 149 patients served for external validation. The prediction performance of random forest (RF), neural network (NN), and extreme gradient boosting (XGBoost) models was compared using comprehensive performance metrics. The Local Interpretable Model-Agnostic Explanations (LIME) methods elucidated the best-performing model. POST incidences across training, validation, and testing cohorts were 41.7%, 38.4%, and 36.2%, respectively. Five predictors were age, sex, endotracheal tube cuff pressure, endotracheal tube insertion depth, and the time interval between extubation and the first drinking of water after extubation. After incorporating these variables, the NN model demonstrated superior generalization capabilities in predicting POST when compared to the XGBoost and RF models in external validation, achieving an area under the receiver operating characteristic curve (AUROC) of 0.81 (95% CI 0.74–0.89) and a precision–recall curve (AUPRC) of 0.77 (95% CI 0.66–0.86). The model also showed good calibration and clinical usage values. The NN model outperforms the XGBoost and RF models in predicting POST, with potential applications in the healthcare industry for reducing the incidence of this common postoperative complication.

## Introduction

Around 313 million surgeries are annually performed worldwide, with the majority of patients undergoing general anesthesia with an endotracheal tube [1]. Postoperative sore throat (POST), described as pain or discomfort in the larynx or pharynx during the postoperative period [2], is a frequent complication that arises following general anesthesia with tracheal intubation, with an incidence between 19% and 62% [3, 4]. Though POST often resolves independently, it can heighten patient dissatisfaction, increase analgesic use, and elevate healthcare costs [2, 5]. Moreover, POST may interfere with the patient’s ability to eat and drink, potentially leading to dehydration, malnutrition, and delayed recovery [6, 7]. In more severe cases, it may exacerbate pre-existing respiratory issues, posing additional challenges for patient management. Thus, it is crucial to develop strategies for the prevention and management of POST to improve the overall patient experience.

Machine learning showcases significant potential in predicting medical outcomes and complications, aiding clinicians in making informed decisions, and enhancing patient care [8–10]. The development of an accurate and reliable prediction model for POST could enable healthcare professionals to identify patients at high risk of experiencing this complication, allowing for the implementation of targeted preventive measures and interventions. Recent advancements in machine learning techniques, from traditional linear models to advanced deep learning architectures, have shown the ability to handle large, heterogeneous datasets and capture intricate relationships among variables that may not be apparent using conventional statistical methods [11–13].

The objective of this study was to develop and validate a machine learning-driven prediction model for POST using a diverse set of patient characteristics and perioperative factors. We aimed to compare the performance of various machine learning algorithms in terms of their predictive accuracy, generalizability, and clinical utility. Furthermore, we evaluated the performance of our model both internally, using a validation cohort from the same institution, and externally, through its application on an independent dataset from another hospital. Ultimately, we seek to provide a valuable tool for clinicians that can assist in the early identification of patients at risk for POST and facilitate the implementation of targeted preventive strategies to reduce the incidence of this common and unpleasant postoperative complication.

## Materials and methods

### Study design

We retrospectively analyzed the medical records of patients admitted to the Affiliated Changzhou Second People’s Hospital of Nanjing Medical University (Jiangsu, China) or the Third Affiliated Hospital of Soochow University (Jiangsu, China). All data were extracted by experienced abstractors, who were blinded to the study hypothesis. To ensure the study’s quality, we adhered to the Strengthening the Reporting of Observational Studies in Epidemiology (STROBE) guidelines [14].

### Patients

We collected data from a series of 961 consecutive adult patients (≥ 18 years) who had an American Society of Anesthesiologists (ASA) physical status ranging from 1 to 3. These patients underwent general anesthesia with endotracheal intubation at either the Affiliated Changzhou Second People’s Hospital of Nanjing Medical University or the Third Affiliated Hospital of Soochow University between September 1, 2021, and May 31, 2022. Exclusions were made for patients who: (1) suffered from a mental disorder; (2) had a recent upper respiratory tract infection; (3) underwent more than one intubation attempt; (4) were transferred to the intensive care unit (ICU) with endotracheal intubation after the operation; or (5) were lost to follow-up.

For our training dataset, we reviewed patient records from the 2021 electronic medical database of the Affiliated Changzhou Second People’s Hospital of Nanjing Medical University. The remaining patients from this hospital comprised a validation cohort. For external validation of our prediction model, we retrospectively extracted data from 149 patients at the Third Affiliated Hospital of Soochow University, from September 1, 2021 to December 30, 2021, ensuring they met the specified inclusion and exclusion criteria.

### Data collection and definition of the outcome

All data were collected by trained research personnel using a standardized data collection form. Data collection encompassed patient demographics, medical history, and pertinent clinical information, such as age, sex, body mass index (BMI), medical conditions (hypertension, diabetes mellitus, chronic gastritis, cancer, coronary heart disease, cerebral infarction, asthma, hyperlipidemia, and smoking history), clinical data, such as ASA status, pneumoperitoneum, surgical position (either supine or non-supine, with non-supine encompassing prone and lateral positions), gastric catheterization, anticholinergic drug use, steroid use (dexamethasone or betamethasone), surgical site (thorax/abdomen or extremities), and endotracheal tube cuff pressure (ETCP) at surgery’s end, measured using the pressure gauge (Hi-Lo Hand Pressure Gauge, VBM Medizintechnik, GmbH, Germany).

Endotracheal tube insertion depth (ETID) denotes the distance from the endotracheal tube’s tip to the incisors or lips, serving as a reference for correct placement within the trachea, thus ensuring that the tube cuff lies below the vocal cords but above the trachea’s bifurcation into the bronchi. Proper ETID recording is vital to prevent complications, such as unintentional extubation or bronchial intubation.

Other collected data included the duration of endotracheal tube placement (DOETP), duration of water deprivation (DOWD), and the time interval between extubation and the first drinking water after extubation (TIBEATFDWAE).

The variables were selected based on a combination of factors. Firstly, prior studies identified these variables as clinically significant predictors of the outcome of interest [4, 15, 16]. Their importance has been consistently emphasized in prior research, making them pivotal for our study. Secondly, our data analysis indicated that these variables were consistently available across our dataset. This ensures a comprehensive analysis without the challenge of significant missing data, enhancing the robustness of our results. Lastly, clinical experts in the domain validated the significance and pertinence of each variable for our study.

The outcome of this study was the presence of POST. It was characterized as patients experiencing any of the following symptoms: dryness and discomfort in the oropharynx without voice changes; sore throat with mild hoarseness; obvious sore throat accompanied by hoarseness and other severe changes; or symptoms too severe to speak. Assessments were conducted on the first postoperative morning (roughly 12–24 h post-extubation) using the POST questionnaire, referencing methods previously detailed [15, 16]. The POST grading was as follows: rating of 0 implied absence of sore throat; level 1, mild sore throat (complained of sore throat only when asked); level 2, moderate sore throat (self-reported sore throat); and level 3, severe sore throat (pain and discomfort in the pharynx that cause hoarseness or vocal change).

### Feature selection

To develop the POST prediction model using the training cohort, we employed a rigorous variable selection approach that prevented data leakage. We began by using a pairwise Pearson correlation matrix to check clinical variables for collinearity, setting a pairwise correlation threshold of *r* > 0.8. From collinear variables, the most clinically accessible were chosen for subsequent analysis. Subsequently, we utilized both the Boruta algorithm [17] and the Least Absolute Shrinkage and Selection Operator (LASSO) algorithm [18] in a two-step process.

The Boruta algorithm, a feature selection technique rooted in random forests (RFs), iteratively assesses the significance of each variable. It does this by comparing its importance to that of its randomly permutated counterparts, thus allowing for the identification of truly relevant predictors by eliminating variables with importance levels comparable to random noise [17]. After applying the Boruta algorithm, a set of significant predictors was obtained.

Next, the LASSO algorithm was employed for additional variable selection. LASSO operates as a regularization method, performing simultaneous variable selection and coefficient determination. It places a constraint on the sum of the absolute values of the model parameters. As a result, certain coefficients are diminished to zero, effectively excluding them from the ultimate model [18]. This step produced another set of significant predictors.

In our final selection, we considered only predictors identified by both the Boruta and LASSO algorithms. This intersection ensured the incorporation of the most relevant and robust variables in the development of our POST prediction model. This combined approach aimed to increase the model’s accuracy and generalizability while reducing the risk of overfitting or including irrelevant predictors.

### Model development and validation

We utilized three machine learning classifiers (extreme gradient boosting [XGBoost], RF, and neural network [NN]) to construct predictive models for POST. These algorithms have been explained elsewhere in detail [19, 20]. A brief summary is presented below.

#### Extreme gradient boosting (XGBoost)

XGBoost is a sophisticated implementation of the gradient boosting algorithm, optimized for speed and performance. The algorithm works by iteratively adding learners (typically decision trees) to a model, where each new tree corrects errors made by the previously trained one. Key hyperparameters include the learning rate, maximum depth of the tree, and the number of trees (boosting rounds). We chose XGBoost for our study due to its adeptness at managing sizable datasets and its established excellence across various prediction tasks. Furthermore, XGBoost offers several advantages like handling missing data, built-in cross-validation, and robustness to overfitting, making it a preferred choice for our analysis [19].

#### Random forest (RF)

Like the XGBoost, RF is an ensemble learning method that operates by constructing multiple decision trees during training and outputs the class that is the mode of the classes of the individual trees for classification problems. Key hyperparameters include the number of trees and maximum depth of the trees [20].

#### Neural network (NN)

The NNs, inspired by the general framework of neurons and neuronal circuitry of the human brain, are a set of algorithms designed to facilitate the passage of information from input nodes to hidden layers, thus optimizing the weights and mapping between input and output layers. NNs were chosen for their ability to model complex non-linear relationships and have demonstrated exceptional performance in numerous tasks. Key hyperparameters for NNs include the number of layers, the number of neurons, activation functions, and among others, the learning rate [20].

To ensure consistency, each model incorporated identical input variables. Subsequently, grid and random hyperparameter searches were employed to ascertain optimal hyperparameters for each model within the training data, utilizing the area under the receiver operating characteristic curve (AUROC) as the primary optimization metric. Upon concluding this process, we assessed model performance using a range of metrics: the area under the precision–recall curve (AUPRC), AUROC, calibration curve, Brier score, and Log Loss. Additionally, we calculated accuracy, sensitivity, specificity, positive predictive value (PPV), and negative predictive value (NPV). The AUROC and AUPRC offered insights into the model’s overall predictive capability. The calibration curve, Brier score, and Log Loss were utilized to gauge the calibration, i.e., the reliability and precision of the predictions. Meanwhile, measures like sensitivity, specificity, PPV, and NPV provided a detailed view of each model’s predictive performance. Complementing the above metrics, decision curve analysis (DCA) [21] was conducted to quantify the net benefit at different threshold probabilities, evaluating the models’ utility in decision making. Finally, Local Interpretable Model-Agnostic Explanations (LIME) [22] facilitated the provision of consistent and locally accurate values for each variable within the best-performing prediction model, further enhancing our understanding of the models’ performance.

### Feature importance

To pinpoint the primary determinant of POST within our patient cohort, we evaluated the significance of each feature in the models using permutation feature importance. This method quantifies the significance of individual features by observing the change in the model’s prediction error upon permuting their values. A feature gains prominence when its permutation diminishes the model’s performance, indicating that the model heavily relies on that specific feature for precise prediction. The importance of a variable within machine learning algorithms, such as XGBoost, RF, and NN models, is ascertained through various factors depending on the algorithm employed. As relative importance does not adhere to a consistent scale, we report the findings using scaled importance. This approach recalibrates the relative importance of a variable in relation to the feature possessing the highest relative importance value, ensuring that the plots are easily interpretable and comparable.

### Sample size calculation

In order to circumvent overfitting and secure enhanced precision in prognostic models, a sufficient sample size is imperative for the construction of predictive frameworks. We use a sample size calculated as *n* ═ 
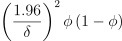
, where φ is the expected outcome ratio (φ ═ 0.40) and δ is the set margin of error (δ ═ 0.05) [23]. As dictated by this formula, the minimal sample capacity for the training set employed in the model’s development amounts to 369 participants. The training population is obviously sufficient for model development.

**Figure 1.**
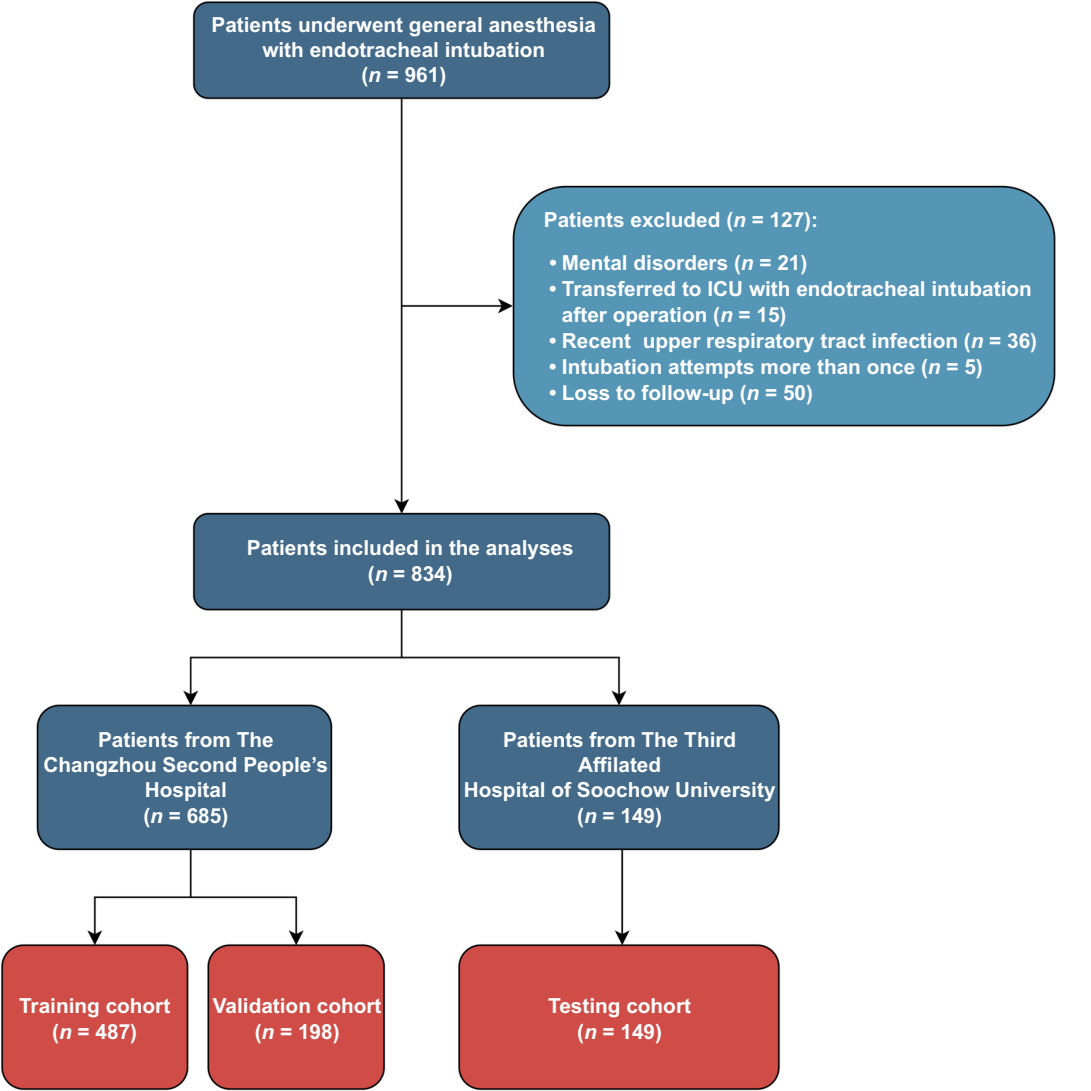
**Flow diagram of patients.** ICU: Intensive care unit.

**Table 1:** Demographic and clinical data of patients

	**All** **(*n* ═ 834)**	**Training cohort** **(*n* ═ 487)**	**Validation cohort** **(*n* ═ 198)**	**Testing cohort** **(*n* ═ 149)**
*Patient characteristics*				
Age (years)	53 (43, 65)	52 (43, 64)	52 (40, 64)	61 (49, 71)
Male	313 (37.5%)	163 (33.5%)	81 (40.9%)	69 (46.3%)
BMI (kg/m^2^)	23.4 (21.4, 25.8)	23.0 (21.2, 25.4)	23.9 (21.9, 26.5)	23.5 (21.8, 26.2)
*Medical history*				
Current smoking	105 (12.6%)	57 (11.7%)	37 (18.7%)	11 (7.4%)
Coronary artery disease	12 (1.4%)	9 (1.9%)	1 (0.5%)	2 (1.3%)
Hypertension	216 (25.9%)	126 (25.9%)	52 (26.3%)	38 (25.5%)
Diabetes mellitus	79 (9.5%)	44 (9.0%)	21 (10.6%)	14 (9.4%)
Hyperlipidemia	8 (1.0%)	5 (1.0%)	2 (1.0%)	1 (0.7%)
Chronic gastritis	3 (0.4%)	3 (0.6%)	0 (0.0%)	0 (0.0%)
Cerebral infarction	12 (1.4%)	5 (1.0%)	2 (1.0%)	5 (3.4%)
Asthma	1 (0.1%)	1 (0.2%)	0 (0.0%)	0 (0.0%)
Cancer	31 (3.7%)	18 (3.7%)	4 (2.0%)	9 (6.0%)
*Procedural characteristics*				
DOWD (hours)	13.8 (11.7, 16.2)	14.5 (11.8, 16.3)	12.3 (10.7, 15.4)	13.8 (11.8, 16.0)
ETID (cm)	22 (21, 23)	22 (21, 23)	22 (22, 23)	21 (21, 23)
ETCP (mmHg)	56 (40, 75)	50 (34, 75)	60 (50, 78)	60 (45, 72)
DOETP (hours)	1.9 (1.3, 2.8)	1.8 (1.3, 2.6)	1.8 (1.2, 2.8)	2.2 (1.6, 2.9)
TIBEATFDWAE (hours)	6.7 (3.0, 15.0)	6.8 (3.2, 15.0)	5.2 (2.4, 15.1)	7.1 (4.6, 14.7)
ASA status				
I-II	786 (94.2%)	471 (96.7%)	191 (96.5%)	124 (83.2%)
III	48 (5.76%)	16 (3.3%)	7 (3.5%)	25 (16.8%)
Position				
Supine	466 (55.9%)	243 (49.9%)	116 (58.6%)	107 (71.8%)
Non-supine	368 (44.1%)	244 (50.1%)	82 (41.4%)	42 (28.2%)
Surgical site				
Thorax or abdomen	721 (86.5%)	436 (89.5%)	160 (80.8%)	125 (83.9%)
Extremities	113 (13.5%)	51 (10.5%)	38 (19.2%)	24 (16.1%)
Pneumoperitoneum	346 (41.5%)	211 (43.3%)	75 (37.9%)	60 (40.3%)
Gastric catheterization	16 (1.92%)	4 (0.8%)	1 (0.5%)	11 (7.4%)
Anticholinergic drug usage	198 (23.7%)	79 (16.2%)	92 (46.5%)	27 (18.1%)
Glucocorticoid usage	712 (85.4%)	396 (81.3%)	176 (88.9%)	140 (94.0%)

Values are presented as the median (interquartile range) or number (percentage). BMI: Body mass index; DOWD: Duration of water deprivation; ETID: Endotracheal tube insertion depth; ETCP: Endotracheal tube cuff pressure; DOETP: Duration of endotracheal tube placement; TIBEATFDWAE: Time interval between extubation and the first drinking water after extubation; ASA: American Society of Anaesthesiologists.

**Figure 2.**
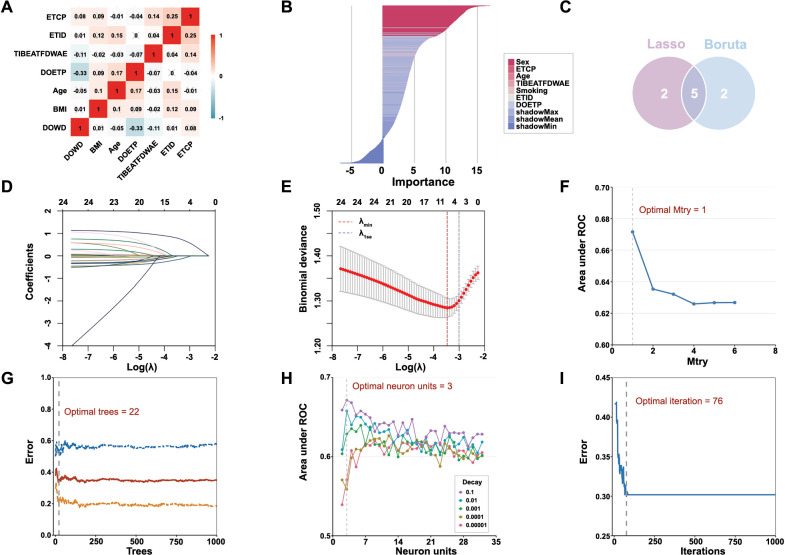
**Selection of variables and model hyperparameters.** (A) Spearman or Pearson correlation matrix of continuous clinical variables. “×” means that the *P* value is less than 0.05, which is not significant. (B) Variable selection by using the Boruta algorithm. (C) Five variables were codetermined by the Boruta and Lasso algorithms. (D and E) Variable selection by using the Lasso regression. (F and G) Determination of optimal hyperparameters for the random forest model. (H and I) Determination of optimal hyperparameters for the neural network model. ETCP: Endotracheal tube cuff pressure; ETID: Endotracheal tube insertion depth; TIBEATFDWAE: Time interval between extubation and the first drinking water after extubation; DOETP: Duration of endotracheal tube placement; BMI: Body mass index; DOWD: Duration of water deprivation; LASSO: Least absolute shrinkage and selection operator.

### Ethical statement

The study protocol was approved by the Institutional Review Boards at the Affiliated Changzhou Second People’s Hospital of Nanjing Medical University (approval number: [KY204-01]) and the Third Affiliated Hospital of Soochow University (approval number: [2023CL036-01]), which waived the need to obtain patient informed consent. This study was conducted in accordance with the principles outlined in the Declaration of Helsinki.

### Statistical analysis

Continuous variables were assessed by using the Shapiro–Wilk test. Those that followed a normal distribution are represented as the mean ± standard deviation (SD). In contrast, non-normally distributed data are depicted as the median with its interquartile range (25th to 75th percentiles). Levene’s test was employed to ascertain the homogeneity of variance across groups. For comparisons of continuous variables between groups, the *t*-test was applied to data with both normal distribution and homogeneity of variance. In instances lacking these attributes, Welch’s *t*-test was employed. For non-normally distributed data, differences were assessed using the Mann–Whitney *U* test. Categorical data are presented as counts and percentages. The significance of differences between groups for categorical variables was determined by the chi-squared test when expected counts were adequate (greater than 5) or Fisher’s exact test for scenarios with low expected counts (less than 5). A two-sided *P* value less than 0.05 was considered statistically significant. All analyses were done with the R software, version 4.1.0.

## Results

### Patient characteristics and incidence

A total of 961 patients were reviewed and 834 patients were included in the study after exclusions had been made, as detailed in Figure 1. These patients were divided into training (487 patients), validation (198 patients), and external validation (149 patients) cohorts. Baseline demographics and perioperative factors across these cohorts are presented in Table 1. The overall incidence of POST in the study population was 39.9%. The incidence of POST in the training, validation, and testing cohorts were 41.7%, 38.4 %, and 36.2%, respectively.

**Table 2:** Comparison of baseline characteristics between patients with and without POST

	**POST** **(*n* ═ 333)**	**Non-POST** **(*n* ═ 501)**	***P* value**
*Patient characteristics*			
Age (years)	50 (40, 61)	57 (45, 68)	**<0.001**
Male	72 (21.6%)	241 (48.1%)	**<0.001**
BMI (kg/m^2^)	23.4 (21.5, 25.6)	23.3 (21.4, 26.0)	0.993
*Medical history*			
Current smoking	82 (16.4%)	23 (6.9%)	**<0.001**
Coronary artery disease	7 (2.1%)	5 (1.0%)	0.238
Hypertension	64 (19.2%)	152 (30.3%)	**<0.001**
Diabetes mellitus	23 (6.9%)	56 (11.2%)	0.052
Hyperlipidemia	2 (0.6%)	6 (1.2%)	0.487
Chronic gastritis	1 (0.3%)	2 (0.4%)	1.000
Cerebral infarction	5 (1.5%)	7 (1.4%)	1.000
Asthma	0 (0.0%)	1 (0.2%)	1.000
Cancer	8 (2.4%)	23 (4.6%)	0.147
*Procedural characteristics*			
DOWD (hours)	13.8 (11.7, 10.6)	13.8 (11.6, 16.2)	0.901
ETID (cm)	21 (21, 23)	22 (21, 23)	**<0.001**
ETCP (mmHg)	60 (42, 82)	50 (38, 70)	**<0.001**
DOETP (hours)	1.8 (1.3, 2.8)	1.9 (1.3, 2.8)	0.958
TIBEATFDWAE (hours)	8.7 (4.0, 16.2)	5.8 (2.8, 14.4)	**<0.001**
ASA status			1.000
I-II	314 (94.3%)	472 (94.2%)	
III	19 (5.7%)	29 (5.8%)	
Position			0.528
Supine	191 (59.4%)	275 (54.9%)	
Non-supine	142 (42.6%)	226 (45.1%)	
Surgical site			0.589
Thorax or abdomen	291 (87.4%)	430 (85.8%)	
Extremities	42 (12.6%)	71 (14.2%)	

Values are presented as the median (interquartile range) or number (percentage). *P* values between groups were assessed by the Chi-square, Fisher’s exact, and Mann–Whitney *U* tests. Bold indicates statistical significance. POST: Postoperative sore throat; BMI: Body mass index; DOWD: Duration of water deprivation; ETID: Endotracheal tube insertion depth; ETCP: Endotracheal tube cuff pressure; DOETP: Duration of endotracheal tube placement; TIBEATFDWAE: Time interval between extubation and the first drinking water after extubation; ASA: American Society of Anaesthesiologists.

### Comparative analysis of POST vs non-POST patients

Differences between patients with and without POST were observed, particularly for age, sex, smoking status, blood pressure, ETCP, and TIBEATFDWAE. Detailed comparisons are available in Table 2.

### Feature selection

All continuous variables showed no pairwise Pearson correlation greater than 0.8 (Figure 2). Using the Boruta and LASSO algorithms, five significant predictors for POST were identified: age, sex, ETCP, ETID, and TIBEATFDWAE (Figure 2). The selected features were incorporated into the three machine learning classifiers (XGBoost, RF, and NN) to develop predictive models for POST.

### Hyperparameter tuning

**Figure S1.**
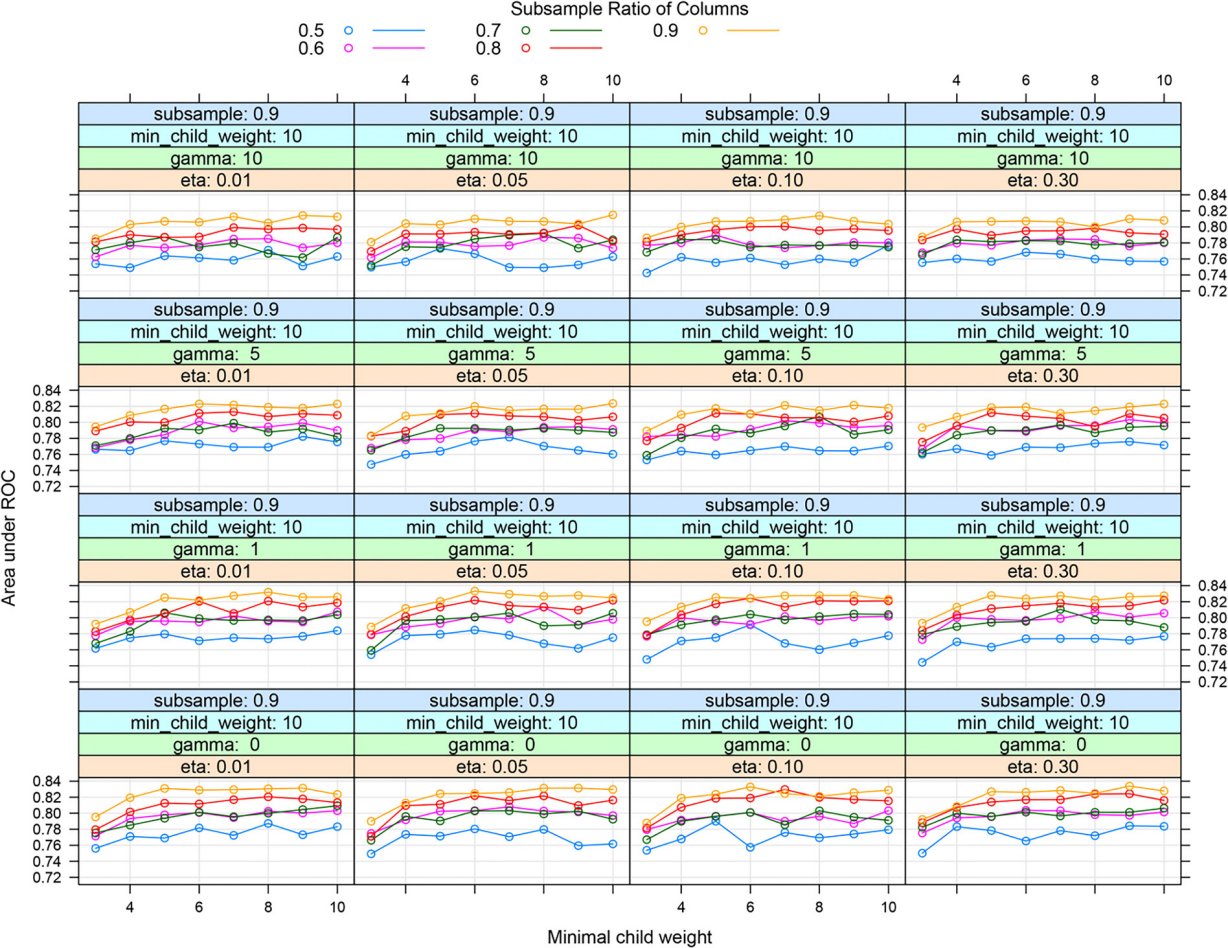
**Grid search method to determine hyperparameters of XGBoost models.** XGBoost: Extreme gradient boosting.

**Figure S2.**
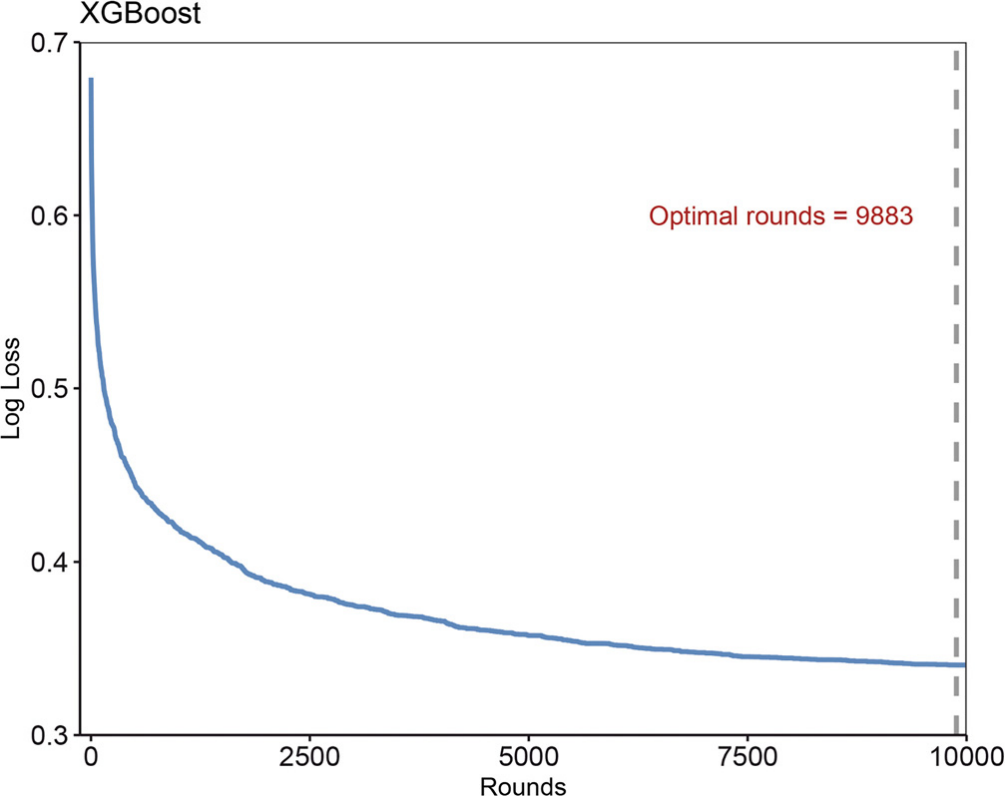
**Grid search method to determine best rounds of XGBoost models.** XGBoost: Extreme gradient boosting.

The process of grid and random hyperparameter searching for RF, NN, and XGBoost algorithms is illustrated in Figures 2F-2I and Figures S1 and S2. The optimal *mtry* and *trees* are 1 and 22, respectively, for RF models. The optimal neural *units*, *decay*, and *iterations* are 3, 0.1, and 76, respectively, for NN models. The optimal *nrounds*, *max_depth*, *eta*, *gamma*, *colsample_bytree*, *min_child_weight,* and subsample were 1, 9, 0.1, 1, 0.8, 10, and 0.9, respectively.

### Model development, validation, and performance

**Figure 3.**
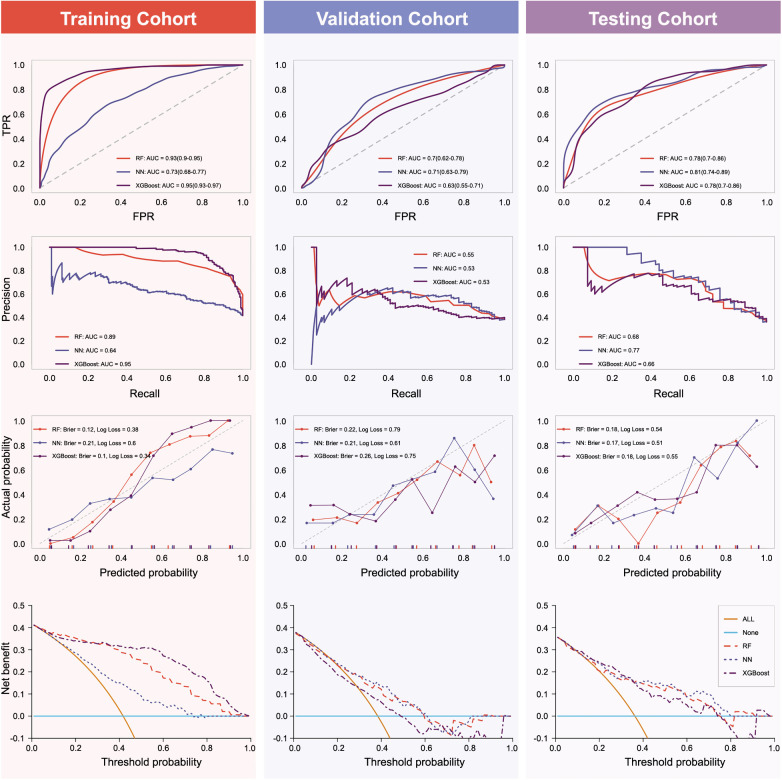
**AUROCs, AUPRCs, calibration plots, and DCA for RF, NN, and XGBoost in the three cohorts**. AUROC: Area under the receiver operating characteristic curve; AUPRC: Area under the precision–recall curve; DCA: Decision curve analysis; RF: Random forest; NN: Neural network; XGBoost: Extreme gradient boosting; TPR: True predictive rate; FPR: False predictive rate.

**Figure 4.**
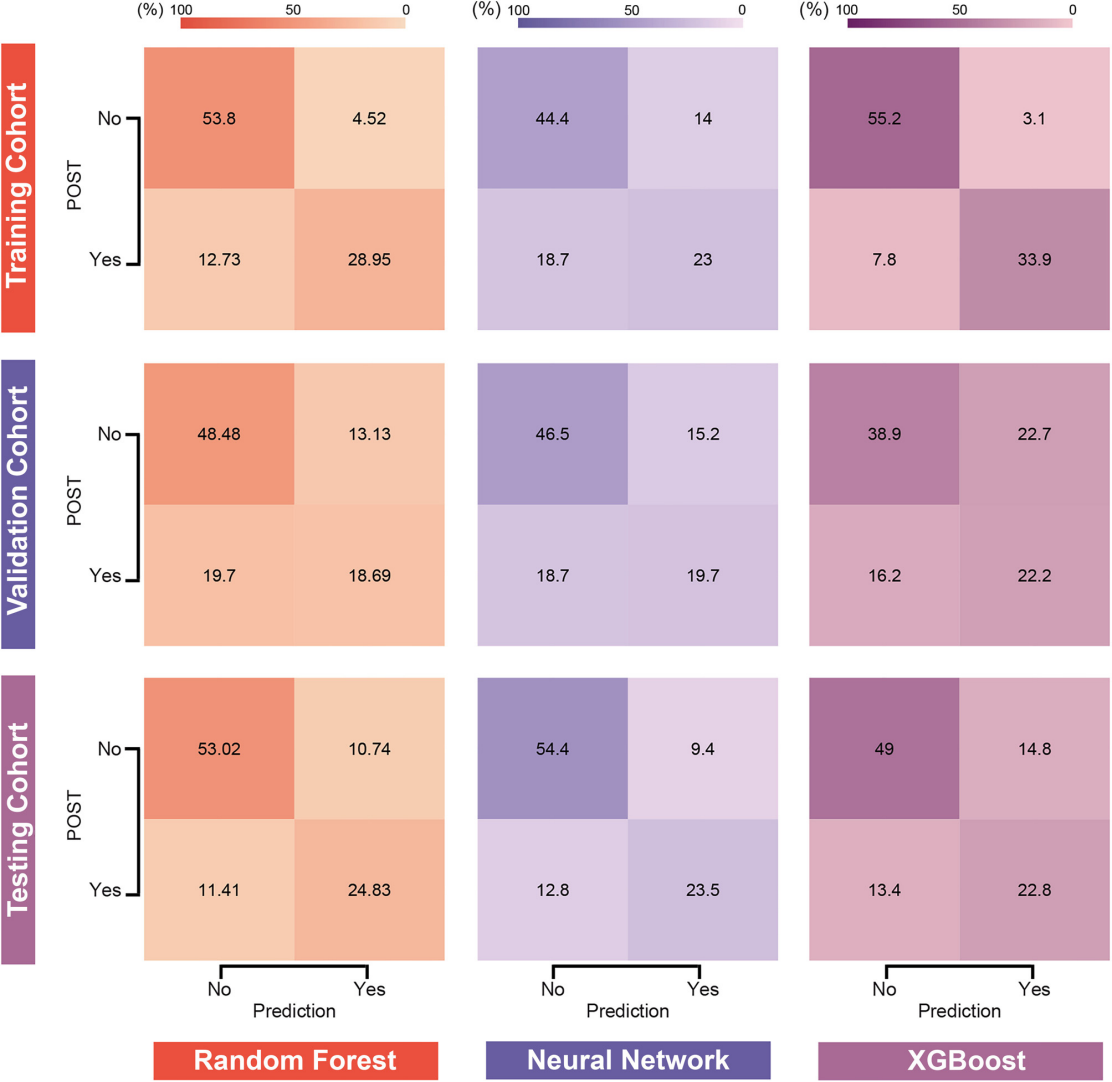
**Confusion matrix plots for RF, NN, and XGBoost models in the three cohorts.** RF: Random forest; NN: Neural network; XGBoost: Extreme gradient boosting; POST: Postoperative sore throat.

Using the identified predictors and optimal hyperparameters, three machine learning models (RF, NN, and XGBoost) were developed and their performances were evaluated. AUROC, AUPRC, Brier scores, and Log Loss metrics were calculated for each model across the training, validation, and external validation cohorts. Figure 3 provides a visual representation, while Figure 4 and Table 3 offer detailed performance metrics.

**Table 3:** Performance metrics for POST prediction models

	**Accuracy (95% CI)**	**Sensitivity (95% CI)**	**Specificity (95% CI)**	**PPV (95% CI)**	**NPV (95% CI)**
*Training cohort*					
Random forest	0.83 (0.79 – 0.86)	**0.94** **(0.89 – 0.97)**	0.78 (0.73 – 0.83)	0.75 (0.69 – 0.85)	**0.94** **(0.91 – 0.96)**
Neural network	0.67 (0.63 – 0.72)	0.69 (0.63 – 0.76)	0.66 (0.60 – 0.71)	0.59 (0.53 – 0.67)	0.75 (0.69 – 0.80)
XGBoost	**0.89** **(0.86 – 0.92)**	0.81 (0.75 – 0.86)	**0.96** **(0.93 – 0.98)**	**0.93** **(0.89 – 0.95)**	0.88 (0.83 – 0.93)
*Validation cohort*					
Random forest	**0.67** **(0.60 – 0.74)**	0.71 (0.60 – 0.81)	0.63 (0.54 – 0.72)	0.55 (0.45 – 0.67)	0.78 (0.68 – 0.84)
Neural network	0.66 (0.59 – 0.73)	**0.72** **(0.61 – 0.82)**	**0.69** **(0.60 – 0.77)**	**0.59** **(0.42 – 0.72)**	**0.80** **(0.70 – 0.86)**
XGBoost	0.61 (0.54 – 0.68)	0.63 (0.51 – 0.74)	0.59 (0.50 – 0.68)	0.49 (0.40 – 0.61)	0.72 (0.61 – 0.79)
*Testing cohort*					
Random forest	**0.78** **(0.70 – 0.84)**	0.67 (0.53 – 0.79)	**0.84** **(0.75 – 0.91)**	**0.71** **(0.58 – 0.82)**	0.82 (0.71 – 0.89)
Neural network	**0.78** **(0.70 – 0.84)**	0.76 (0.62 – 0.87)	0.77 (0.67 – 0.85)	0.65 (0.53 – 0.79)	0.85 (0.75 – 0.90)
XGBoost	0.71 (0.64 – 0.79)	**0.89** **(0.77 – 0.96)**	0.56 (0.45 – 0.66)	0.53 (0.43 – 0.77)	**0.90** **(0.79 – 0.93**)

Bold indicates statistical significance. PPV: Positive predictive value; NPV: Negative predictive value; XGBoost: Extreme gradient boosting; POST: Postoperative sore throat.

### Clinical utility

DCA indicated varying net benefits of the models across different cohorts, suggesting specific clinical utility scenarios for each model (Figure 3). In the training cohort, the DCA showed that the XGBoost’s net benefits for predicting POST exceeded those of RF, NN, and the strategies of treating all or none of the patients when the threshold probability surpassed 27%. However, the RF and NN models showed higher net benefits compared to the XGBoost model in the validation cohort. In the testing cohort, the NN model was superior to the other two, having net benefits ranging from 60% to 80%.

### Feature importance

**Figure 5.**
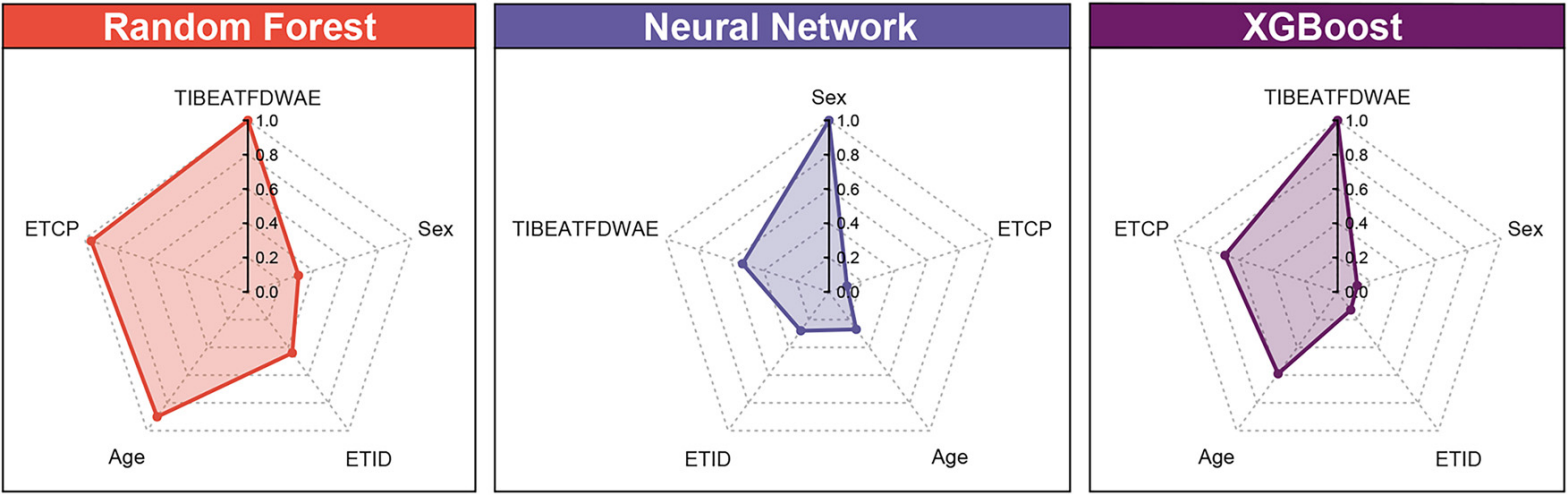
**Relative importance radar plots of five predictors in RF, NN, and XGBoost models:** (A) RF; (B) NN; (C) XGBoost. RF: Random forest; NN: Neural network; XGBoost: Extreme gradient boosting; TIBEATFDWAE: Time interval between extubation and the first drinking water after extubation; ETID: Endotracheal tube insertion depth; ETCP: Endotracheal tube cuff pressure.

Permutation feature importance analysis revealed that the top two important features were TIBEATFDWAE and ETCP in the RF and XGBoost models. Somewhat differently, the two most important predictors in the NN model were sex and TIBEATFDWAE (Figure 5). This indicates that the TIBEATFDWAE variable may have significant ramifications for the POST.

### Model explainability

**Figure 6.**
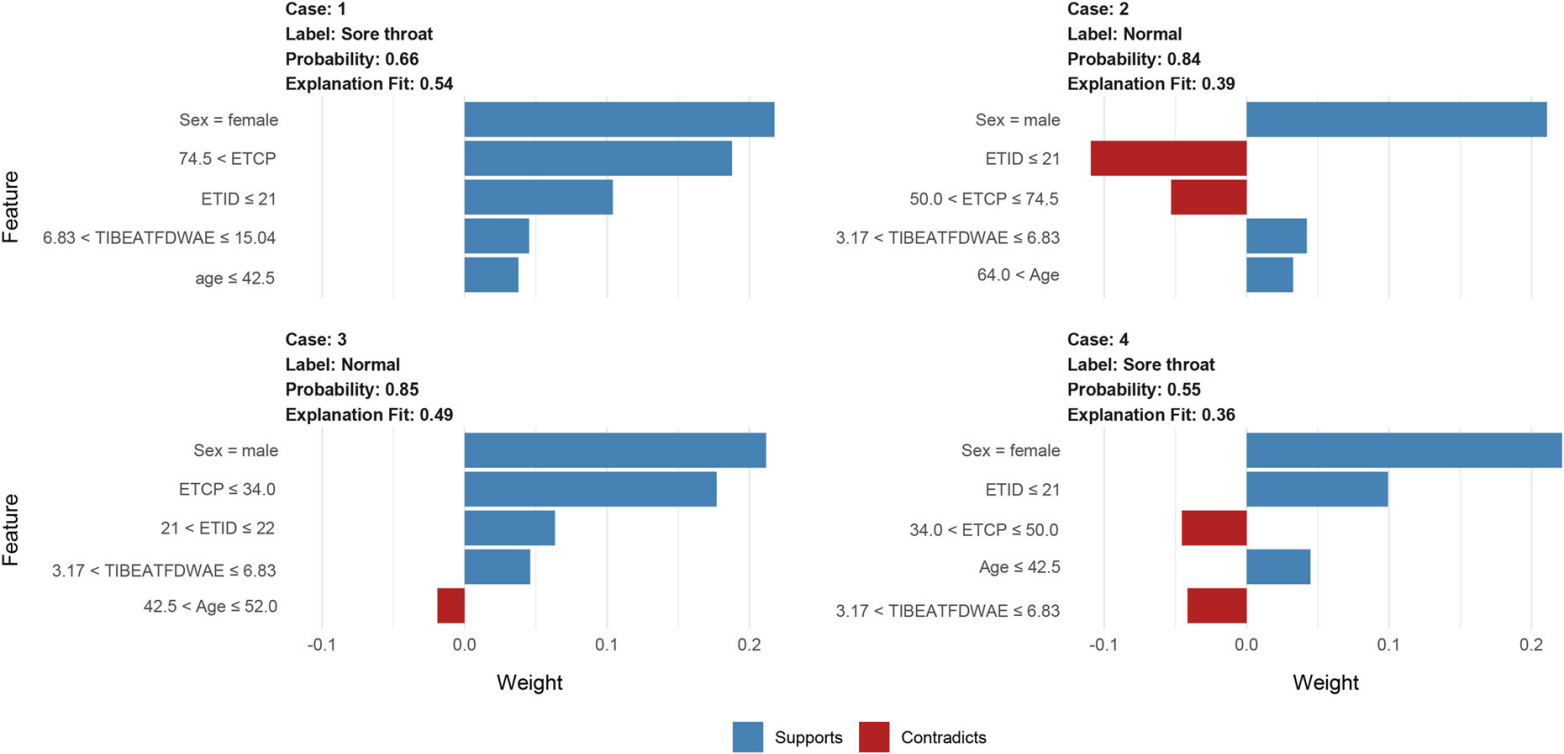
**Interpretation of the neural network with a local interpretable model explainer in four cases.** Two sore throat patients (A, D) and two normal patients (B, C) are illustrated. Features with a blue bar favor the outcome, and those with a red bar contradict the outcome. The *x*-axis shows how much each feature added or subtracted to the final probability value for the patient (i.e., a feature with a weight of 0.3 is equivalent to a 30% change in the probability of the outcome). ETCP: Endotracheal tube cuff pressure; ETID: Endotracheal tube insertion depth; TIBEATFDWAE: Time interval between extubation and the first drinking water after extubation.

Using LIME, we clarified the predictions of the NN model, highlighting how different features influence predictions. We detailed four representative cases to illustrate the interplay of factors in determining POST risk (Figure 6). In case 1, the figure indicated a moderate probability of POST at 66%. All features of this patient were favorable. This might suggest that a younger female patient with a higher ETCP, an extended TIBEATFDWAE, and an improper ETID is more likely to have a POST. For case 2, the NN model predicted a POST probability of only 16%. The interpretative algorithm indicated factors like older age and male gender, coupled with a shorter TIBEATFDWAE lean toward a non-POST outcome. However, an inappropriate ETID and a higher ETCP were negative prognostic factors for this result. Likewise, case 3 had a similar low POST prediction at 15%. Factors, such as age, male sex, a lower ETCP, a proper ETID, and a shorter TIBEATFDWAE all acted as protective attributes, suggesting a non-POST outcome. For case 4, the model assigned a POST probability of 55%. Favorable characteristics, such as female sex, young age, and a shallower ETID tended the algorithm toward POST; however, a lower ETCP and a shorter TIBEATFDWAE were both negative prognostic factors for POST.

## Discussion

### Principal findings

This study aimed to develop and evaluate machine learning models—RF, NN, and XGBoost—for predicting POST in patients undergoing surgery with endotracheal intubation. To the best of our knowledge, this may be the first predictive model capable of assessing the risk of POST for patients intubated after general anesthesia. Our findings indicated that the XGBoost model outperformed the RF and NN models in the training cohort. However, in the validation cohort, the RF and NN models exhibited higher net benefits than the XGBoost model. In the external testing cohort, the NN model surpassed the other two within a particular range of net benefits. This study offers valuable insights into POST prediction, enriching existing knowledge and serving as a foundation for subsequent research and potential clinical applications.

Our prediction models identified five crucial predictors for POST: age, sex, ETCP, ETID, and TIBEATFDWAE. These predictors are acknowledged risk factors for POST in the literature. Age and sex are well recognized as demographic risk factors for POST, with younger patients and females having a higher risk [24–26]. Consistent with previous research, ETCP emerged as a significant predictor for POST [27]. Generally, after a successful tracheal intubation, the anesthesiologist inflates the tracheal tube cuff to ensure optimal ventilation and minimize anesthetic leakage. The ETCP often depends on the anesthetist’s expertise and manual balloon palpation, typically surpassing the recommended ETCP of 15–25 mmHg [28, 29]. Excessive ETCP can hamper the blood flow to the tracheal mucosa, leading to issues like ischemia, ulceration, and necrosis of the tracheal mucosa, hence causing throat discomfort [27]. Consistent with other reports, the ETID is another significant predictor for POST [4, 24, 27]. Biro et al. [24] observed that the incidence of POST increased with increasing duration of endotracheal intubation. The TIBEATFDWAE aligns with existing studies linking the DOWD to POST incidence [30–32]. Traditionally, patients receiving general anesthesia were allowed to drink water about 4–6 h after awakening from anesthesia (for non-gastrointestinal surgery) to ensure postoperative safety and prevent coughing, vomiting, and aspiration caused by oral hydration [33]. However, with the advent of Enhanced Recovery after Surgery concepts, many studies validated the benefits of early oral hydration (roughly an hour post-anesthesia awakening), citing reduced thirst and oropharyngeal discomfort [30–32]. In addition, pre-anesthesia gargling with licorice may alleviate POST [34, 35]. Our findings are consistent with these previous studies, emphasizing the association between the time interval from extubation to first water intake and POST incidence.

In the training cohort, the XGBoost model achieved the highest AUROC and AUPRC values, indicating superior predictive performance compared to RF and NN models. The model also demonstrated good calibration and lower Brier scores and Log Loss values, suggesting better prediction accuracy. However, in the validation cohort, the RF and NN models showed higher net benefits compared to the XGBoost model. The discrepancies in performance between the training and validation cohorts could stem from the XGBoost model’s overfitting. Overfitting occurs when a model performs well on the training data but fails to generalize to unseen data [36].

The NN model demonstrated superior performance within a certain range of net benefits in the external cohort. This suggests that the NN model may be more suitable for clinical use in specific scenarios. The DCA performed in this study helps determine the clinical usefulness of the models by quantifying the net benefits at different threshold probabilities. Such DCA findings can assist clinicians in selecting the ideal model based on the precise clinical setting and their preferred risk threshold [21].

### Strengths

This study represents a series of significant advancements in the ongoing efforts to predict and alleviate POST. Uniquely, it pioneers the utilization of machine learning algorithms for POST prediction, moving beyond the traditional statistical methods that have predominated prior research. While earlier studies have illuminated various POST predictors, our work extends the scope by exploring less conventional variables, such as the TIBEATFDWAE. The inclusion of both internal and external validation cohorts in our analysis not only underscores the robustness of our findings but also extends an invitation for their broader applicability across varied clinical settings. Clinically, the derived predictive models stand to transform patient care by empowering clinicians with insights to identify and proactively manage individuals at an elevated risk for POST, aiming for enhanced postoperative patient comfort and satisfaction. Through this innovative approach, we aspire for our study to serve as a cornerstone for future research, fostering further exploration and refinement of machine-learning-based predictive models in the realm of postoperative complications.

### Limitations

Several limitations of this study should be considered. First, the sample size may be insufficient for thorough validation of the models, possibly affecting the generalizability of the findings. Furthermore, data were collected retrospectively, which could introduce potential biases and confounders. Future studies with larger sample sizes and prospective data collection can help address these limitations and further validate the predictive performance of the models. Moreover, the study did not consider certain factors that may influence POST, such as the use of different airway devices, lubrication, and the anesthetist’s experience. Including these factors in future research may enhance the predictive capabilities of the models. Lastly, the study did not assess the impact of implementing the models in clinical practice on patient outcomes and resource utilization. Future research should investigate the potential benefits of incorporating these models in clinical decision making, such as the reduction of POST incidence and the optimization of resource allocation.

## Conclusion

The present study demonstrates that the NN model outperforms the XGBoost and RF models in predicting POST. This superior model has the potential to aid healthcare professionals in identifying patients at high risk for POST, thereby facilitating the implementation of targeted preventive strategies and ultimately reducing the incidence of this common and unpleasant postoperative complication.

## Supplemental data

## Data Availability

The original contributions presented in the study are included in the article and additional files. Further data that support the findings of this study are available from the corresponding author upon reasonable request.

## References

[1] Meara JG, Leather AJ, Hagander L, Alkire BC, Alonso N, Ameh EA (2015). Global surgery 2030: evidence and solutions for achieving health, welfare, and economic development. Lancet.

[2] Hailu S, Shiferaw A, Regasa T, Getahun YA, Mossie A, Besha A (2023). Incidence of postoperative sore throat and associated factors among pediatric patients undergoing surgery under general anesthesia at Hawassa University comprehensive specialized hospital, a prospective cohort study. Int J Gen Med.

[3] Park SY, Kim SH, Lee SJ, Chae WS, Jin HC, Lee JS (2011). Application of triamcinolone acetonide paste to the endotracheal tube reduces postoperative sore throat: a randomized controlled trial. Can J Anaesth.

[4] El-Boghdadly K, Bailey CR, Wiles MD (2016). Postoperative sore throat: a systematic review. Anaesthesia.

[5] Aqil M, Khan MU, Mansoor S, Mansoor S, Khokhar RS, Narejo AS (2017). Incidence and severity of postoperative sore throat: a randomized comparison of Glidescope with Macintosh laryngoscope. BMC Anesthesiol.

[6] Reber E, Gomes F, Vasiloglou MF, Schuetz P, Stanga Z (2019). Nutritional risk screening and assessment. J Clin Med.

[7] Porter K, Burch N, Campbell C, Danbury C, Foster C, Gabe S (2021). Supporting people who have eating and drinking difficulties. Clin Med.

[8] Jayatilake S, Ganegoda GU (2021). Involvement of machine learning tools in healthcare decision making. J Healthc Eng.

[9] Giordano C, Brennan M, Mohamed B, Rashidi P, Modave F, Tighe P (2021). Accessing artificial intelligence for clinical decision-making. Front Digit Health.

[10] Javaid M, Haleem A, Pratap Singh R, Suman R, Rab S (2022). Significance of machine learning in healthcare: features, pillars and applications. Int J Intell Netw.

[11] Rajula HSR, Verlato G, Manchia M, Antonucci N, Fanos V (2020). Comparison of conventional statistical methods with machine learning in medicine: diagnosis, drug development, and treatment. Medicina.

[12] Ley C, Martin RK, Pareek A, Groll A, Seil R, Tischer T (2022). Machine learning and conventional statistics: making sense of the differences. Knee Surg Sports Traumatol Arthrosc.

[13] Choudhary K, DeCost B, Chen C, Jain A, Tavazza F, Cohn R (2022). Recent advances and applications of deep learning methods in materials science. NPJ Comput Mater.

[14] von Elm E, Altman DG, Egger M, Pocock SJ, Gotzsche PC, Vandenbroucke JP (2007). Strengthening the reporting of observational studies in epidemiology (STROBE) statement: guidelines for reporting observational studies. BMJ.

[15] Chen Z, Jin Y, Lu G, Jin Y, Feng C, Zhao X.

[16] Niu J, Hu R, Yang N, He Y, Sun H, Ning R (2022). Effect of intratracheal dexmedetomidine combined with ropivacaine on postoperative sore throat: a prospective randomised double-blinded controlled trial. BMC Anesthesiol.

[17] Speiser JL, Miller ME, Tooze J, Ip E (2019). A comparison of random forest variable selection methods for classification prediction modeling. Expert Syst Appl.

[18] Vasquez MM, Hu C, Roe DJ, Chen Z, Halonen M, Guerra S (2016). Least absolute shrinkage and selection operator type methods for the identification of serum biomarkers of overweight and obesity: simulation and application. BMC Med Res Methodol.

[19] Kalyani P, Manasa Y, Ahammad SH, Suman M, Anwer TMK, Hossain MA (2023). Prediction of patient’s neurological recovery from cervical spinal cord injury through XGBoost learning approach. Eur Spine J..

[20] Kunze KN, Polce EM, Sadauskas AJ, Levine BR (2020). Development of machine learning algorithms to predict patient dissatisfaction after primary total knee arthroplasty. J Arthroplasty.

[21] Sadatsafavi M, Adibi A, Puhan M, Gershon A, Aaron SD, Sin DD (2021). Moving beyond AUC: decision curve analysis for quantifying net benefit of risk prediction models. Eur Respir J.

[22] Sathyan A, Weinberg AI, Cohen K (2022). Interpretable AI for bio-medical applications. Complex Eng Syst.

[23] Stevenson MA (2020). Sample size estimation in veterinary epidemiologic research. Front Vet Sci.

[24] Biro P, Seifert B, Pasch T (2005). Complaints of sore throat after tracheal intubation: a prospective evaluation. Eur J Anaesthesiol.

[25] Higgins PP, Chung F, Mezei G (2002). Postoperative sore throat after ambulatory surgery. Brit J Anaesth.

[26] Levin PD, Chrysostomos C, Ibarra CA, Ledot S, Naito D, Weissman C (2017). Causes of sore throat after intubation: a prospective observational study of multiple anesthesia variables. Minerva Anestesiol.

[27] Liu J, Zhang X, Gong W, Li S, Wang F, Fu S (2010). Correlations between controlled endotracheal tube cuff pressure and postprocedural complications: a multicenter study. Anesth Analg.

[28] Svenson JE, Lindsay MB, O’Connor JE (2007). Endotracheal intracuff pressures in the ED and prehospital setting: is there a problem?. Amer J Emerg Med.

[29] Sole ML, Penoyer DA, Su X, Jimenez E, Kalita SJ, Poalillo E (2009). Assessment of endotracheal cuff pressure by continuous monitoring: a pilot study. Amer J Crit Care.

[30] Yin X, Ye L, Zhao L, Li L, Song J (2014). Early versus delayed postoperative oral hydration after general anesthesia: a prospective randomized trial. Int J Clin Exp Med.

[31] Yin X, Zeng X, Wang T, Dong B, Wu M, Jia A (2020). Early versus delayed postoperative oral hydration in children following general anesthesia: a prospective randomized trial. BMC Anesthesiol.

[32] Mercan A, El-Kerdawy H, Bhavsaar B, Bakhamees HS (2011). The effect of timing and temperature of oral fluids ingested after minor surgery in preschool children on vomiting: a prospective, randomized, clinical study. Paediatr Anaesth.

[33] Watcha MF, White PF (1992). Postoperative nausea and vomiting. Its etiology, treatment, and prevention. Anesthesiology.

[34] Agarwal A, Gupta D, Yadav G, Goyal P, Singh PK, Singh U (2009). An evaluation of the efficacy of licorice gargle for attenuating postoperative sore throat: a prospective, randomized, single-blind study. Anesth Analg.

[35] Ruetzler K, Fleck M, Nabecker S, Pinter K, Landskron G, Lassnigg A (2013). A randomized, double-blind comparison of licorice versus sugar-water gargle for prevention of postoperative sore throat and postextubation coughing. Anesth Analg.

[36] Rhyou SY, Yoo JC (2023). Aggregated micropatch-based deep learning neural network for ultrasonic diagnosis of cirrhosis. Artif Intell Med.

